# Genome-wide discovery of lincRNAs with spatiotemporal expression patterns in the skin of goat during the cashmere growth cycle

**DOI:** 10.1186/s12864-018-4864-x

**Published:** 2018-06-26

**Authors:** Shen Song, Min Yang, Yefang Li, Marhaba Rouzi, Qianjun Zhao, Yabin Pu, Xiaohong He, Joram M. Mwacharo, Ning Yang, Yuehui Ma, Lin Jiang

**Affiliations:** 1 State Key Laboratory of Animal Nutrition, Institute of Animal Science (IAS), Chinese Academy of Agricultural Sciences (CAAS), Beijing, 100193 China; 2 Department of Animal Genetics and Breeding, China Agricultural University, Beijing, 100094 China; 3 Small Ruminant Genomics Group, International Center for Agricultural Research in the Dry Areas (ICARDA), P. O. Box 5689, Addis Ababa, Ethiopia

**Keywords:** Goat skin, lincRNA, Hair follicle, Transcriptome, Cashmere growth cycle

## Abstract

**Background:**

Long intergenic noncoding RNAs (lincRNAs) have been recognized in recent years as key regulators of biological processes. However, lincRNAs in goat remain poorly characterized both across various tissues and during different developmental stages in goat (*Capra hircus*).

**Results:**

We performed the genome-wide discovery of the lincRNAs in goat by combining the RNA-seq dataset that were generated from 28 cashmere goat skin samples and the 12 datasets of goat tissues downloaded from the NCBI database. We identified a total of 5546 potential lincRNA transcripts that overlapped 3641 lincRNA genes. These lincRNAs exhibited a tissue-specific pattern. Specifically, there are 584 lincRNAs expressed exclusively in only one tissue, and 91 were highly expressed in hair follicle (HF). In addition, 2350 protein-coding genes and 492 lincRNAs were differentially expressed in the skin of goat. The majority exhibited the remarkable differential expression during the transition of the goat skin from the May–June to August–October time point, which covered the different seasons. Fundamental biological processes, such as skin development, were significantly enriched in these genes. Furthermore, we identified several lincRNAs highly expressed in the HF, which exhibited not only the co-expression pattern with the key factors to the HF development but also the activated expression in the August to October time point. Intriguingly, one of spatiotemporal lincRNAs, *linc-chig1598* could be a potential regulator of *distal-less homeobox 3* expression during the secondary hair follicle growth.

**Conclusions:**

This study will facilitate future studies aimed at unravelling the function of lincRNAs in hair follicle development.

**Electronic supplementary material:**

The online version of this article (10.1186/s12864-018-4864-x) contains supplementary material, which is available to authorized users.

## Background

Transcripts longer than 200 nucleotides and lacking coding capability are defined as long noncoding RNA (lncRNA) [1, 2]. Based on its relative location to the neighbouring protein-coding genes, lncRNA can be classified into four categories: long intergenic noncoding RNA (lincRNA), intronic lncRNA, antisense lncRNA, and enhancer RNA [1, 3]. The lincRNA comprises lncRNA genes in the intergenic region lacking known protein-coding genes. Thousands of lincRNAs have been discovered through large-scale cDNA collections [4], genome-wide tilling microarrays [5, 6], high-throughput RNA sequencing (RNA-seq) [7–11], and histone modification mapping [12] in animals and plants [4, 6]. Despite their non-coding nature, lncRNAs play key roles in diverse biological processes, such as cell cycle regulation [12], transcriptional regulation [13, 14], pluripotency maintenance [15], and development [16]. More than 15,000 human [17] and 10,000 mouse [18] lincRNAs have been identified to date. Interestingly, mammalian lncRNAs exhibited greater tissue specificity than protein coding genes [9]. Spatiotemporal-specific lncRNAs have been discovered in several tissues of macaque and mouse brain during organ development [19, 20]. Therefore, detecting lincRNAs with spatiotemporal expression is essential for characterizing and understanding the functions and molecular mechanisms of lncRNAs during development.

The domestic goat (*Capra hircus*) provides a major source of meat, milk, fibre, and fur for humans. Thus, it plays a key role in agro-industry, socio-cultural, religious and economics of, particularly developing countries across Asia and Africa, which contain 95% of the world’s goat population. As one of the most important domestic goat breeds, Cashmere goat, which is double-coated for both wool and cashmere, is famous for its fine fibre production traits. Two types of hair follicles (HFs) exist in the Cashmere goat skin, the primary hair follicles (PHFs) for wool and the secondary hair follicles (SHFs) for cashmere [21]. The growth and regeneration of SHFs recurs annually throughout the lifetime of each goat [22]. The SHF cyclic process involves several phases of growth (early anagen, April–August; anagen, August–November), regression (catagen, December–January), and quiescence (telogen, February–March) [23, 24]. Early studies of mouse HFs noted that the ability to propagate anagen induction is limited to early anagen follicles. When the propagating early anagen follicles have progressed into late anagen, the propagation does not resume [25]. Thus, the traditional anagen period was divided into early propagating and late autonomous anagen, with respectively low and high expression of inhibitors, such as bone morphogenetic proteins (BMPs) [25, 26]. However, whether there is a clear transition from the early anagen phase to the anagen phase of SHFs in goat and which factor is crucial for the transition remain unclear. A transcriptome analysis of fetal and postnatal skeletal muscle identified 3981 goat lncRNAs, including 3515 lincRNAs and 466 antisense lncRNA [27]. Another study identified 2943 goat lncRNAs during puberty and they speculated that many of these lncRNAs are involved in the regulation of puberty and reproduction trait in goat [28]. A recent study detected the expression of 1366 lncRNAs (including 999 lincRNAs) in the skin of foetal goat and noted the strict tissue specificity and functional conservation of several lncRNAs during skin development and pigmentation [29]. However, caprine lincRNAs have not yet been systematically investigated across diverse tissues and during the growth phases of the HFs at the genome-wide scale. Thus, few lincRNAs have been identified to have spatiotemporal expression and co-expression with the key HF factors during SHF growth phases.

Recent advances in whole-transcriptome sequencing technology and the availability of the goat reference genome provide an opportunity to comprehensively annotate the caprine lincRNAs and characterize the lincRNAs with spatiotemporal expression. Here, we performed an integrative analysis of the existing large-scale RNA-seq data sets to systematically discover lincRNAs in goat. In addition, we profiled the expression of these lincRNAs across ten different tissues to detect the expression pattern of lincRNAs. Moreover, we compared the expression changes of the lincRNAs at five developmental time-points of the SHFs to identify time-specific lincRNAs. Finally, we analyzed the co-expression clusters, performed a gene ontology (GO) enrichment analysis and overlapped time-specific lincRNAs with the tissue-specific lincRNAs to predict the biological function of the lincRNAs with spatiotemporal expression during SHF growth. Our study facilitates the further exploration of the fundamental function of lincRNAs during the development stages in skin, a particularly important organ in Cashmere goat.

## Results

### Genome-wide detection and characterization of lincRNAs expressed in diverse tissues of goat

To comprehensively detect the lincRNAs in goat, we combined all available goat RNA-seq datasets from the NCBI (http://www.ncbi.nlm.nih.gov/, see Materials and Methods, Additional file 1: Table S1). These 12 downloaded datasets covered the majority of the caprine organs or tissues (e.g., heart, liver, lung, skin, muscle, kidney, and brain) and are suitable for obtaining a comprehensive view of goat lincRNAs (Additional file 1: Table S1). We developed a computational pipeline for goat lincRNA identification, which mainly comprised the transcriptome reconstruction and filtering process (Additional file 2: Figure S1).

For transcriptome reconstruction, greater than 2.7 billion RNA-seq reads from 74 samples, including 28 skin samples generated by our team, were mapped to the goat reference genome [30] using TopHat [31] and then subject to the assembly software Cufflinks [32] to construct the caprine transcriptome. We classified all transcripts that did not overlap the known protein-coding genes using Cuffcompare [32], and 958 to 148,174 transcripts were identified in the intergenic regions in each sample (Additional file 1: Table S1). The lincRNA transcripts often exhibit low expression and are minimally distinguishable from the abundant assembled fragments with low expression and reliability. Thus, we removed the unreliable transcripts with FPKM (fragments per kilobase of transcript per million mapped reads) values less than 0.5. For each analysis, the output transcripts that were located at least 500-bp away from any known protein-coding genes were further merged by Cuffmerge [32], resulting in 246 to 4081 transcripts for 74 samples analyzed here (Additional file 1: Table S1). Subsequently, we calculated the coding potential of the remaining transcripts using the Coding Potential Calculator (CPC) tool [33], predictor of long non-coding RNAs and messengers RNAs based on an improved k-mer scheme (PLEK, 2ersion 1.2) [34], and Coding Non-Coding Index (CNCI, version 2) software [35]. The transcripts which identified as coding transcript by PLEK and with CPC score > 0 and CNCI score > 0 were discarded. Finally, 7472, 8635, and 8184 transcripts were identified as reliable lincRNA transcripts by CPC, CNCI, and PLEK, respectively. The transcripts which identified by the intersection results from these three softwares were combined into 5546 potential lincRNA transcripts that overlapped 3641 lincRNA genes (Additional file 1: Table S1).

**Fig. 1:**
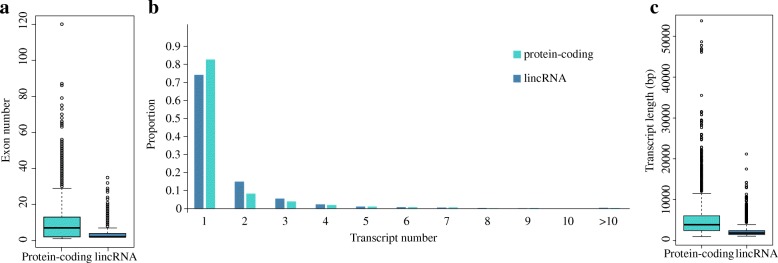
Genomic structure characteristics of lincRNAs compared to protein-coding genes. **a**. Distribution of exon number for predicted lincRNAs and protein-coding genes. **b**. Distribution of the number of transcripts in the predicted lincRNAs and protein-coding genes. **c**. Distribution of the transcript lengths of the predicted lincRNAs and protein-coding genes

We subsequently characterized the basic genomic features of the obtained lincRNAs and compared these features with the available features of the goat protein-coding genes. Given that fewer lincRNAs (*N* = 3641) than protein-coding genes (*N* = 27,834) were identified, an equal number of protein-coding genes were randomly selected to enable a comprehensive comparison of the genomic features. As a result, the majority of the predicted lincRNAs contained two to three exons (mean: 3.27), which is considerably lower than the number of the protein-coding genes (mean: 9.64, Fig. 1a). The mean number of alternatively spliced transcripts for lincRNAs and protein-coding genes is approximately 1.52 (Fig. 1b). Consistent with the lower number of exons, the length of the lincRNAs was typically less than 2000 bps (mean: 1324 bp), which was notably shorter than the length of protein-coding genes (4007.76 bp, Fig. 1c). The genomic features of the caprine lincRNAs were consistent with those of lincRNAs detected in other species.

A recent transcriptome analysis of the fetal goat skin (100-d) identified 999 lncRNA transcripts [29]. Based on the sequence-based comparative analysis (see methods), we found 630 (11.36%) transcripts (corresponding to 308 lincRNA genes) overlapped with the lincRNA set identified in the previous report.

### Tissue-specific expression of the detected lincRNAs in goat

**Fig. 2:**
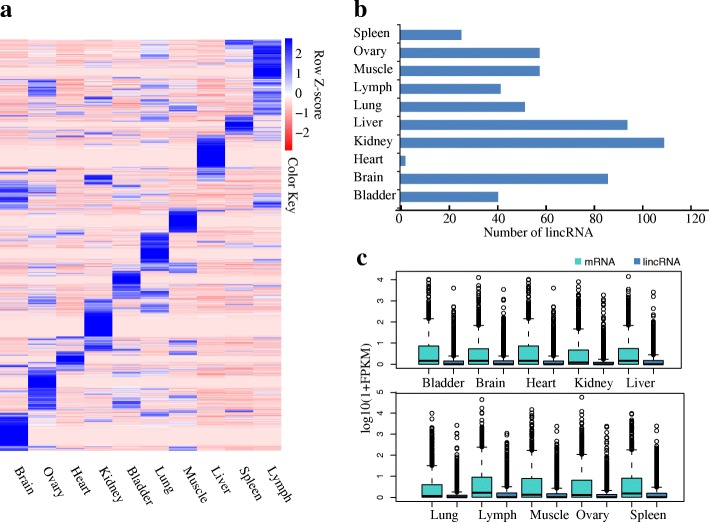
Tissue-wide distributions and expression level of lincRNAs. **a**. Heatmap of 5546 lincRNAs across 10 tissues. Each row represents the expression levels of all detected lincRNAs, and each column contains all expressed transcripts. We transformed the FPKM values into the log2 (FPKM+ 1) values and then calculated the Z-score for every log 2 (FPKM+ 1) value within each tissue. **b**. Histograms indicating the number of tissue-specific lincRNAs in each tissue. **c**. Expression level indicated by log10 (FPKM+ 1) in lincRNAs and protein-coding genes in each tissue

We used the RNA-seq datasets for ten different tissues types from the same goat (GSE37456) to characterize tissue-specific expression pattern of the detected lincRNAs. The FPKM value was calculated to examine the distribution of the gene expression level for each lincRNA in each sample. Of the 3641 predicted lincRNA gene, 2721 were expressed in the ten-tissue panel. A heatmap based on the expression pattern in all tissues was generated across all expressed lincRNAs. Interestingly, the majority exhibited a tissue preferential expression pattern (Fig. 2a), with 2137 lincRNAs expressed in more than one tissue. The remaining 584 lincRNAs displayed exclusive expression in one tissue, with the most tissue-specific lincRNAs being in the kidney (*N* = 108) and the least tissue-specific lincRNAs being in the spleen (*N* = 25) (Fig. 2b). The average expression level of lincRNAs is reduced compared with that of protein-coding genes in all available tissues (Kolmogorov-Smirnov (KS) test *P* < 2E-16, Fig. 2c). Subsequently, we analysed the co-expression pattern across ten tissues for the lincRNAs and their proximal protein-coding genes within a 10-kb distance. A total of 2063 protein-coding gene-lincRNA expression pairs which covered 1472 lincRNA genes were observed. Interestingly, these protein-coding gene-lincRNA pairs were more correlated with the expression pattern than the random and proximal protein-coding gene-protein-coding gene pairs (KS test, *P* < 2E-16), suggesting the co-regulation and functional association of lincRNAs and the proximal protein-coding genes (Additional file 3: Figure S2).

### lincRNA expression during SHF cycling in skin

**Fig. 3:**
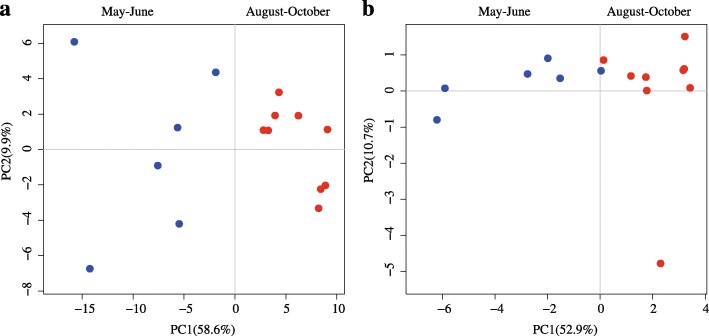
PCA of all expressed genes and lincRNAs. PCA based on all genes: all differentially expressed genes (**a**) and PCA based on the lincRNAs: differentially expressed lincRNAs (**b**). Blue dots represent May and June, whereas red dots represent Aug, Sep, and Oct

To investigate the expression profiling of lincRNAs in skin, a particularly important organ for cashmere goat, we used the RNA-seq data generated from 15 skin samples at five SHF developmental time points (May, June, August, September, and October) in the cashmere growth phase (early anagen, April–August; anagen, August–November). Using the Illumina Hiseq2500 platform, we generated a total of 156.77, 179.85, 210.50, 173.35, and 156.85 million 125-bp paired-end reads for samples at the May, June, August, September, and October time points, respectively (Additional file 4: Figure S3A), with approximately 90% of the clean reads matching the reference goat genome in TopHat [31]. Subsequently, principal component analysis (PCA) of all samples from five time points exhibited notable separation between May–June (blue dot) and August–October (red dot) based on the expression of either genes (Fig. 3a) or lincRNAs (Fig. 3b). It is possible that a remarkable transcriptional difference occurred between the different seasons, which consistent with the early anagen and anagen phases of hair follicle development.

**Fig. 4:**
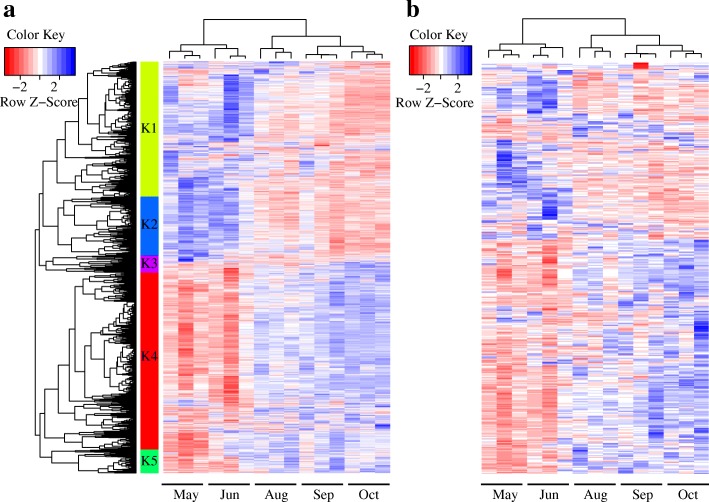
Hierarchical clustering of all differentially expressed genes (**a**) and lincRNAs (**b**). We transformed the RPKM values into the log10 (FPKM+ 1) values and then calculated the Z-score for every log10 (FPKM+ 1) value within each tissue. The five main clusters (K1-K5) are presented on the left panel **a**

To further illustrate the significant differences in the temporal expression of both lincRNAs and protein-coding genes across different seasons, we used the criteria of at least two-fold differences in the FPKM values and a *q* value of less than 5% for at least one of ten pair-wise comparisons of the five time points. Overall, 2350 protein-coding genes and 492 lincRNAs are differentially expressed between the ten comparisons. In addition, hierarchical clustering was conducted using the expression of protein-coding genes and lincRNAs during the May–June to August–October time points. Both all differentially expressed genes (including protein-coding genes and lincRNA, Fig. 4a) and lincRNA (Fig. 4b) expression could distinguish the two groups: May–June and August–October. The majority of time-specific mRNAs (1663/2350) and lincRNAs (461/492) exhibited differential expression between the May–June and August–October time points (Additional file 4: Figure S3B). The relative expression levels for seven protein-coding genes and 12 lincRNAs across all development time points were measured by RT-qPCR to validate these time-specific lincRNAs and protein-coding genes. Although the expression data for a few lincRNAs, such as those of lincRNA *linc-chig3050* and *linc-chig1715*, were associated with slightly low R values across the time points tested, the trend of regulation was consistent between these two tests. The excellent correlation between the RNA-Seq and RT-qPCR validated the reliability gene expression quantification by RNA-seq (Additional file 5: Figure S4). Therefore, our findings revealed that similar to protein-coding genes, lincRNAs exhibited notable expression changes in skin along with the seasons, and possibly highlighting their key roles in the transition from the early anagen phase to the anagen phase.

### Function prediction of lincRNAs based on co-expression clusters and spatiotemporal expression

A total of 2350 differentially expressed protein-coding genes and 492 lincRNAs generated in our dataset, were selected for hierarchical clustering to identify co-expression clusters. The co-expression analysis resulted in the identification of five distinct clusters (K1-K5). The expression change patterns for the five clusters are presented in Fig. 4a, and the genes assigned to each cluster are provided in Additional file 6: Table S2. These five clusters contained 558, 58, 769, 1114, and 343 genes, including 77, 21 92, 216, and 86 lincRNAs, respectively (Fig. 4). Two main expression patterns were identified in both protein-coding genes and lincRNAs. Clusters K1-K3 represent the down-regulated gene clusters, whereas clusters K4 and K5 represent the up-regulated gene clusters (Fig. 4a). We performed GO enrichment analysis to reveal the over-represented biological processes associated with each cluster using g:profiler [36].

**Fig. 5:**
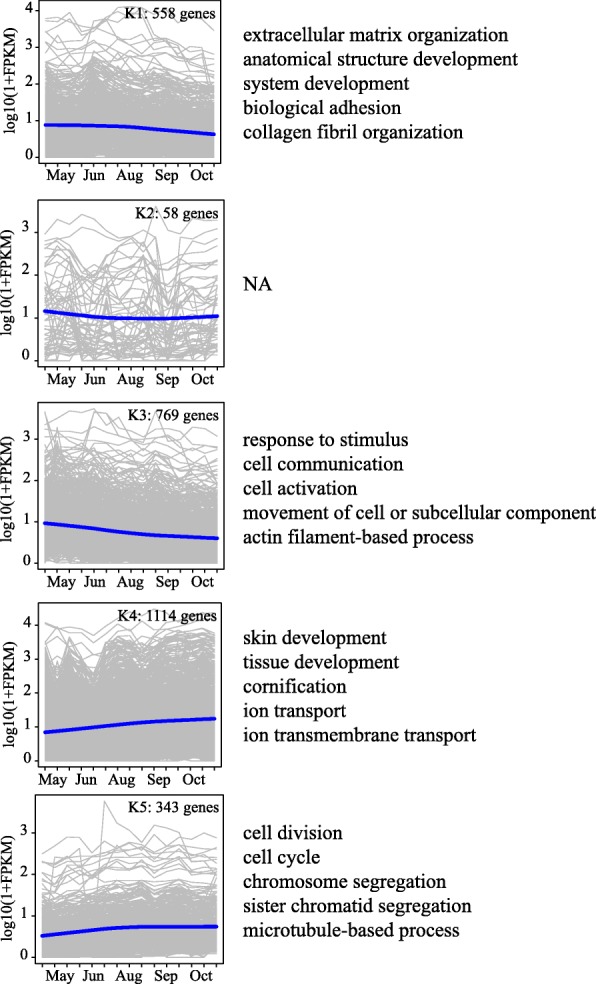
Expression patterns of the differentially expressed genes (including protein-coding genes and lincRNAs) in the five main clusters (K1-K5) corresponding to the Fig. 4a and the significantly enriched GO terms in different clusters

The results showed that genes related to extracellular matrix organization, biological adhesion, and collagen fibril organization were clustered into cluster K1, and genes linked to response to stimulus, cell communication, cell activation, movement of cell or subcellular component and actin filament-based process were clustered into cluster K3 (Additional file 6: Table S2). Interestingly, cluster K4 exhibited a significant over-representation of functional relevant GO categories/pathways, such as hair development, the tissue development and the cornification pathway (Fig. 5, Additional file 6: Table S2). Many identified genes of the K4 clusters have been reported as a key regulator of HF differentiation and cycling in human or mouse, such as *BARX2* [37], *FOXN1* [38], *OVOL1* [39], and *VDR* [40–42], *DLX3* [43], *HOXC13* [44, 45], *MSX2* [46, 47], and *MSX1* [48] in cluster K4. This result suggested that the activated expression of these genes during the transition from the May–June to August–October time point may be crucial to HF growth in Cashmere goat. In addition, GO term related to cell division, cell cycle, chromosome segregation, sister chromatid segregation and microtubule-based process were over-represented in the cluster K5. Genes in these functional categories include several genes that are important for cell proliferation, such as *CDC6* and *CDCA3*. In addition, 16 lincRNA-mRNA co-expression pairs were defined (Additional file 7: Table S3), indicating the possible regulatory role of these lincRNAs in skin to adapt to the change of seasons.

We overlapped the temporal lincRNAs from the identified co-expression clusters with the HF highly expression lincRNAs that were identified in the PHF and SHF RNA-seq datasets (GSE37456) (Additional file 8: Table S4). 91 lincRNAs were found highly expressed in the HF, 51 of which showed differentially expressed. Interestingly, 47 spatiotemporal lincRNAs with upregulation in HFs belong to the cluster K4 and K5 (Additional file 7: Table S3). Among these 47 lincRNAs, four were in the vicinity of the HF development-related genes and two were near the keratin-associated proteins (Additional file 7: Table S3) and one (*linc-chig*1598) was close to two transcription factors, *DLX4* and *DLX3*. The DLX transcription factors play critical roles in epidermal, neural and osteogenic cellular differentiation, and DLX3 is a crucial regulator of HF differentiation and cycling [43, 49]. Both *DLX3* and *DLX4* exhibited a significant positive correlation with the lincRNA *linc-chig1598* in our own RNA-seq data set (*linc-chig1598* vs. *DLX4*, Pearson’s test, *p* = 2.101e-05, *R* = 0.87; *linc-chig1598* vs. *DLX3*, Pearson’s test, *p* = 8.986E-08, *R* = 0.95) and RT-qPCR experiment (*linc-chig1598* vs. *DLX4*, Pearson’s test, *p* = 0.0174, *R* = 0.94; *linc-chig1598* vs. *DLX3*, Pearson’s test, *p* = 4.77E-3, *R* = 0.97). When compared the *linc-chig1598* to the human genome in UCSC, a homologous lincRNA RP11-304F15.6 (ENSG00000254039.1) to *linc-chig1598* is located at the upstream region of the human *DLX3/DLX4* gene locus. These results suggested that *linc-chig1598* is conserved between different species, and may be a potential regulator of *DLX3* expression during the SHF growth phases of goat.

## Discussion

In this study, we discovered a set of 3641 caprine lincRNA transcripts using a bioinformatics pipeline and ~ 675 G RNA-seq data in diverse tissue and cell types. As expected, these lincRNAs displayed a clear tissue-preferential expression pattern. Among these lincRNAs, we identified a catalogue of 584 tissue-specific lincRNAs exclusively in only one tissue, indicating their fundamental functions in the corresponding tissues. In addition, more than two thousand time-specific lincRNAs and protein-coding genes were identified in the skin tissue of Cashmere goat, which exhibited divergent expression patterns between May–June and August–October time point. Many genes related to the SHF growth exhibit differential expression. GO enrichment analysis indicated that these genes could be involved in the fundamental functions, such as hair development, in the SHF of goat. Furthermore, we found four spatiotemporal HF highly expression lincRNAs that exhibited not only co-expression with the key factors for HF development but also activated expression during the transition from the May–June to the August–October time point, which covered the early anagen phase and the anagen phase of SHF development. Intriguingly, one spatiotemporal lincRNAs, *linc-chig*1598, was perfectly co-expressed with a crucial regulator of HF differentiation and cycling *DLX3*, suggesting that it could be a potential regulator of *DLX3* expression during SHF growth.

Given that only 20% of transcripts are non-poly adenylated [50], thousands of lincRNAs in animal and plant were discovered through the poly(A) RNA-seq [9, 51], suggesting that poly(A) RNA-seq is highly effective for the discovery of lincRNAs. Therefore, we used poly(A) RNA-seq data that were publicly available and also generated by our group, designed a pipeline based on previous reports [9, 52], and discovered more than 5000 lincRNA transcripts in various tissues and cell types of goat. These lincRNAs had a shorter length, fewer exons, and fewer alternative splice transcripts and were expressed at a lower level than protein-coding genes, which is consistent with the findings in human [52], mouse [53], zebrafish [16], and pig [11]. Almost 11.36% of the identified lincRNAs (or genes) in our study were already discovered in a recent skin RNA-seq study [29]. This relatively small percentage of the overlap might be due to the difference in various aspects of the two studies, such as sampled tissue types and developmental stages. In addition, reproducible detection of lincRNAs in the RNA-seq analysis and RT-qPCR validation indicated that they are bona fide transcripts rather than the random products from the transcriptional noise (Additional file 5: Figure S4). Finally, the perfect validation of the time-specific expression pattern of the lincRNAs by RT-qPCR further supported the accuracy of gene expression quantification by RNA-seq.

Studies in human [9], pig [11], and rainbow trout [54] showed that the number of tissue-specific lincRNAs is increased in the kidney, liver, and brain, suggesting the expression of tissue-specific lincRNAs in association with certain biological processes in specific tissue. The co-expression analysis of lincRNAs with the neighbouring protein-coding genes showed that the correlation between lincRNAs and protein-coding genes is higher than both the neighbouring coding gene pairs and random coding gene pairs (Additional file 3: Figure S2). This finding is consistent with the previous findings which showed that lincRNAs have a greater likelihood to be functionally associated with their nearest neighbouring protein-coding genes [55, 56]. In other words, the function of unknown lincRNA genes can be possibly predicted by their protein-coding gene neighbours.

In addition to tissue-specific expression, we observed time-specific expression of the caprine lincRNAs during the developmental stages of a special tissue in goat, namely, skin. The skin of Cashmere goat contains thousands of primary and secondary HFs. The SHF undergoes yearly regenerative cycling through phases of growth (early anagen and anagen), regression (catagen), and quiescence (telogen) [57, 58]. At the base of this cycle is the ability of HF cells to briefly exit their quiescent status to generate transient amplifying progeny. However, how these cells are activated during the commencement of anagen remains largely unclear. In the present study, we measured the expression of both lincRNAs and protein-coding genes across five time points in different season. Both PCA and hierarchical clustering analysis of the dynamic expression of lincRNAs and protein -coding genes exhibited a transition point between the end of June and the beginning of August, which respectively covered the stage of early anagen and anagen stage of SHF growth. This indicates that the time point June to August may be a key point for SHF development. Thus, by comparing samples before and after the transition point, we identified 2350 time-specific genes, including 492 lincRNAs, and the majority were up-regulated at the start of August. These findings indicated the up-regulated expression of these lincRNAs and protein-coding genes may closely associate with the activation of HF cells or reflect the seasonal effects on the skin.

The time-specific genes were grouped into five gene clusters by hierarchical clustering. GO enrichment analysis of each gene cluster revealed significant over-representation of skin/tissue development and cornification in two major clusters (K4 and K5) with up-regulated expression during the transition from May–June to August–October time point. The results indicate the possible regulatory role of lincRNA in cluster K4 to skin development in Cashmere goat. Previous studies in mouse and rabbit reported that activated expression of inhibitors, such as BMPs, and high expression of Wnt signalling proteins can trigger the HFs into the anagen phase [25, 26]. In our study, the periodic expression of genes in the BMP signalling pathway, including *TGFB1* and *TGFBR3*, and the high expression of Wnt signalling proteins, including *WNT2*, *WNT11*, *WNT16*, and *LEF1*, indicated the conservation of the regulatory mechanism during HF growth. We also identified the up-regulation of several homeobox transcription factors key to HF development and differentiation, including *DLX3* [43], *HOXC13* [44, 45], *MSX2* [46, 47], and *MSX1* [48], during the transition to the anagen phase from the early anagen phase. In addition, genes in cluster K4 were significantly enriched in the GO categories of skin/tissue development and cornification. These results indicated the possible regulatory role of these lincRNAs in goat skin during May–June to August–October time point. Interestingly, by overlapping with follicle-specific lincRNAs, we identified a catalogue of spatiotemporal lincRNAs that were co-expressed with the factors key to the development of HFs, including *linc-chig275*, *linc-chig1296*, *linc-chig343*, and *linc-chig1598*. One spatiotemporal lincRNA, *linc-chig1598*, displayed positively correlated expression with its neighbour genes, *DLX3* and *DLX4*. Due to the central role of DLX3 in HF morphogenesis, formation, differentiation and cycling programs [43, 59], *linc-chig1598* may have a regulatory role in the expression of *DLX3/DLX4* loci during the growth of SHFs in goat. Therefore, additional studies, such as loss of function experiments or epigenetic analysis, are needed in the future to provide further insights on the regulatory function of *linc-chig1598* in HFs.

## Conclusions

This study provides a catalog of goat lincRNAs, and will facilitate future studies aimed at unravelling the function of lincRNAs in hair follicle development.

## Methods

### Sample collection and experimental design

Experimental cashmere goats were obtained from Inner Mongolia, China. All cashmere goats were raised by feeding practices according to the Cashmere goat standard. The adult individuals (female) were randomly selected. Genetic relationships were avoided during the sampling process. Skin samples were collected from the scapular region from 28 goats at five consecutive time points throughout the year, including May 1st, June 7th, August 7th, September 8th, and October 28th. For each goat, after hair shearing and alcohol deiodination, approximately 1 cm^2^ of skin tissue was grasped with sterile forceps and quickly cut near the tip using sterile scalpel blades. Each clipping was obtained immediately adjacent to the location of the previous shearing. Yunnan Baiyao powder (Yunnan Baiyao Group Co., Ltd., China) was applied immediately to stop the bleeding. For each piece of skin tissue, half was stored in RNAlater (Thermo Fisher Scientific, USA) and then stored at − 80 °C until RNA extraction, and the other half was stored in 4% paraformaldehyde fixation solution to prepare paraffin sections. All experimental procedures involving the cashmere goats used in this study were approved by the Animal Care and Use Committee of the Ministry of Agriculture of the People’s Republic of China. Another two RNA-seq datasets from our lab, Bange and Liaoning Cashmere goat, generated from goat mixed tissues were also used in the present study (Additional file 1: Table S1).

### RNA extraction, quality analysis and sequencing

Total RNA was extracted from the collected skin tissues using the RNeasy Mini kit (Qiagen) following the manufacturer’s instructions. The RNA concentration and quality were further determined using an Agilent 2100 Bioanalyzer. Samples with an RIN (RNA integrity number) value greater with 8.0 were used for sequencing. The mRNA selection, library preparation and sequencing were performed at the BerryGenomics Company (Beijing, China) on an Illumina HiSeq2500 sequencer. The cDNA library for RNA-seq was prepared using the Illumina TruSeq RNA library Preparation Kit v2 following the company’s instructions. Briefly, after the end-repair and adding of poly(A) to the 3’end of the RNA fragments, the sequencing linker was ligated and the ligation products were purified using for PCR amplification. The PCR products were separated by 2% agarose gel electrophoresis and the 400-500 bp fragments were selected to construct sequencing libraries. Finally, the libraries were sequenced on a Hiseq2500 (Illumina) using 150 × 2 bp paired-end sequencing strategy.

### Public data sources

Goat genome assembly CHIR_1.0 (10 September 2015) and the GFF3 file were downloaded from NCBI (http://www.ncbi.nlm.nih.gov/). The set of data, which included 12 RNAseq datasets, was downloaded from the NCBI SRA database. Detailed information regarding the RNA-seq datasets was provided in Table S1.

### Mapping and assembly of transcriptomic data and lincRNA identification

For lincRNA discovery, we used the skin transcriptome data generated from 28 skin samples of cashmere goat (Additional file 1: Table S1) by this study and the 12 downloaded RNAseq datasets derived from 46 goat samples with different tissue-types or cell types. Quality filtering of the raw reads was performed using NGSQC Toolkit v2.3.3 [60]. For both single-end and pair-end library, the raw reads that contained ambiguous bases were filtered; then, we discarded reads having an overall quality score below 20 (with flag -s 20) using the PHRED33 scale for at least 70% (with flag -l 70) of the bases and any bases with a quality score below 20 at 3′ end of the reads. For paired-end library, only paired reads were used for further analysis.

The clean reads were mapped to the goat genome using TopHat version 2.1.0 with flag -g [31]. The mapped reads for each sample were assembled using Cufflinks (v2.2.1) with flag -g [32]. To identify long intergenic non-coding RNAs, novel transcripts or genes were identified by comparing the assembled transcriptomes from each sample to the goat reference transcriptome GTF file using Cuffcompare [32]. Transcripts which assigned with a class code ‘u’ were selected as putative novel transcripts. Next, we used the following exclusion criteria to obtain high-confidence transcripts: 1) transcripts with single-exon lincRNAs were excluded; 2) unreliable transcripts with a FPKM value less than 0.5 in each sample were excluded; 3) transcripts should be 500 bps away from the protein coding region; 4) transcripts with length less than 200 bps were excluded. The pipeline used to identify putative lincRNAs from the RNA-seq data is presented in Additional file 1: Figure S1. After filtering, the assembled transcript fragments of lincRNAs from the all samples for each study were merged into a single GTF file using the tool CuffMerge. Finally, transcripts with a CPC [33] score > 0, CNCI (version 2) [34] score > 0 and transcripts with category “coding” assigned by PLEK (version 1.2) [35] were excluded for each study. The putative lincRNAs were merged to create a consensus lincRNA transcriptome GTF file, and the consensus lincRNA transcriptome was merged with the protein coding gene annotations using the tool Cuffcompare to obtain a consensus novel transcript GTF (Additional file 9: Table S5) for further analysis.

We performed a sequence-based comparative analysis of the lincRNA set identified in our study with the one found in a recent study [29] by using a BLASTn search (blastn -query lincRNA.fasta -out RH -db RH -evalue 1e-5 -outfmt 7). We only considered the transcripts with less than three mismatches, larger than 98% sequence identity, less than six gaps open and longer than 200-bp transcript. In addition, we compared the *linc-chig*1598 to the human genome in UCSC (http://genome.ucsc.edu/) with the default setting.

### Tissue-specific identification of lincRNAs

We use RNA-seq data sets from ten tissues (lymph, lung, spleen, kidney, liver, muscle, heart, brain, bladder, and ovary) from the Yunling black goat and HF samples from the cashmere goat (GSE37456) [30] to characterize the expression pattern of the lincRNA genes. We used the aforementioned consensus GTF file. The clean reads were mapped to the goat genome using TopHat version 2.1.0 with flag -G. The FPKM value was calculated using Cufflinks with flag -G to examine the distribution of the gene expression level. The heatmap was generated using all expressed lincRNA gene in ten tissues using the pheatmap package in R/Bioconductor (https://cran.r-project.org/web/packages/pheatmap/index.html) based on the FPKM values. The level of gene expression is visualized by using a colour gradient from red to blue. For ten tissues from Yunling black goat, lincRNAs were classified into tissue-specific lincRNAs that were observed in only one tissue. Highly expressed lincRNA in hair follicle was defined as the lincRNA with average FPKM value of 20-fold greater than of other 10 tissues, and the average FPKM value of hair follicle should at greater than 2.5-fold than in other tissues. After filtering, a total of 91 lincRNAs met our criteria and were cited as HF highly expressed lincRNAs (Additional file 8: Table S4). We defined lincRNAs and their proximal protein-coding genes within a 10-kb distance as lincRNA-protein-coding gene pair.

### Identification and expression profiling of lincRNA candidates in skin during the HF cycle

Three individuals (5c7317, 5c8418 and 5e5107 for May; 6c02419, 6c8418 and 6c7327 for June, 8c02419, 8c8418 and 8c7327 for August, 9c02419, 9c8418 and 9c7327 for September and 10c02419, 10c8418 and 10c7327 for October, Additional file 1: Table S1) for each time points were selected to determine the expression profile in skin during the five developmental time-points. Differential expression of coding genes and lincRNA across time points was then estimated using the Cuffdiff software [32]. The differential expression changes were calculated for the pairwise comparison between all the time points. Genes with a fold change of ≥2 and a corrected *P* value of ≤0.05 were considered differentially expressed. Gene showing significantly differential expression pattern in at least one comparison was defined as the time-specific gene. Principal component analysis (PCA) was performed using the k-means method to cluster samples based on the FPKM value in R (*rgl* package). All statistical analyses were performed using R software (https://www.r-project.org/).

### Cluster analysis

Genes that were differentially expressed between at least one pair of time points in anyone of the five time points were likely related to the HF development. The log10-fold changes in expression among different time points were obtained for a total 2350 protein-coding genes and 492 lincRNAs. Hierarchical clustering analyses were conducted using hierarchical cluster function hclust from base package stats of R and complete linkage and Pearson correlation measures were used to establish clusters.

### GO enrichment analysis

GO functional enrichment analysis was performed using G:Profiler to identify the GO functional categories enriched during HF development [34]. The default settings were used, and GO terms with a significant *P* value (*P* value < 0.05) after multiple testing correction through G:Profiler’s G:SCS method were considered.

### Validation of RNA-seq data by reverse transcription quantitative real-time PCR (RT-qPCR)

Seven protein-coding genes and 11 lincRNAs were randomly selected for RT-qPCR analysis to assess the expression patterns deduced from the sequencing data. We performed additional RT-qPCR experiment to validate the RNA-seq data using the same samples which used for RNA-seq. The expression levels of the selected protein-coding genes were normalized against that of β-actin. The expression levels of the selected lincRNAs were normalized against that of three different reference genes, *SDHA*, *UBC* and *YWHAZ* [61]. The primers for RT-qPCR are listed in Additional file 10: Table S6. One microgram of RNA from each sample was reversed transcribed to cDNA using a RT-PCR kit (TaKaRa, Dalian, China). The RT-qPCR mixture contained 2 μl of a 1:20 dilution of the cDNA from each samples, primers for a final concentration of 0.4 μM each, 10 μl of the SYBR® Premix Ex TaqTM II (TaKaRa, Dalian, China) and 6.8 μl ddH_2_O up to a total volume of 20 μl. The qPCR was performed under the following cycling conditions: 95 °C 30 s, and 40 cycles of 5 s at 95 °C and 34 s at 60 °C in 96-well optical reaction plates. The qPCRs were performed on an ABI7500 (Applied Biosystems, USA). Sample cycle threshold (Ct) values were determined and standardized relative to the reference control, and the 2^-ΔΔCt^ method was used to calculate the relative changes in gene expression. qPCR was conducted in triplicate. Correlations of relative RT-qPCR and RNA-seq data were performed using the Pearson correlation coefficient method.

## Additional files

Additional file 1: **Table S1**. Details on the RNA-seq datasets. (XLSX 162 kb)

Additional file 2: **Figure S1**. Overview of the informatics pipeline used to the identify lincRNAs in goat. (PDF 216 kb)

Additional file 3: **Figure S2**. Density plot of Pearson correlation coefficient. (PDF 151 kb)

Additional file 4: **Figure S3**. Characterization of the HF development-related gene expression in skin. Note: A. Number of RNA-seq reads obtained from the 15 samples at five time points. B. Histogram presenting differentially expressed genes with an adjusted *P* value ≤0.05 for at least one of the ten comparisons (May vs. Jun, May vs. Aug, May vs. Sep, May vs. Oct, Jun vs. Aug, Jun vs. Sep, Jun vs. Oct, Aug vs. Sep, Aug vs. Oct, and Sep vs. Oct). (PDF 275 kb)

Additional file 5: **Figure S4**. Comparison of the expression patterns of selected lincRNAs and protein-coding genes detected in RNA-seq (red line) and RT-qPCR (blue line) assays revealing a high correlation between the two methods. Note: The log2 ratios of the expression changes during Oct relative to the other time points were calculated and plotted (the ratio was set to 0 for the normal condition). The expression levels of the selected protein-coding genes were normalized against that of *β-actin*. The expression levels of the selected lincRNAs were normalized against that of *SDHA, UBC* and *YWHAZ*. The R values (Pearson correlation coefficients) across the different time points are presented for each gene. (PDF 543 kb)

Additional file 6: **Table S2**. Gene function enrichment analysis based on the GO annotation of DEGs. (XLSX 56 kb)

Additional file 7: **Table S3**. Cluster of differential expressed lincRNAs and their proximal genes. Blank in yellow represents hair follicle development-related gene; lincRNA ID in bold represent the differentially expressed HF-highly expressed lincRNAs. (XLSX 43 kb)

Additional file 8: **Table S4**. FPKM values of lincRNA which show highly expression in HF of goat. (XLSX 59 kb)

Additional file 9: **Table S5**. GTF file for goat lincRNAs. (XLSX 813 kb)

Additional file 10: **Table S6**. Primer sequences for RT-qPCR. (XLSX 55 kb)

## Abbreviations

FPKM: Fragments Per Kilobase of exon per million fragments mapped
GO: Gene Ontology
HF: Hair follicle
lincRNA: Long intergenic noncoding RNA
PCA: Principal component analysis
PHF: Primary hair follicle
SHF: Secondary hair follicle
