# Supplementary figures and images for: Genome-wide discovery of lincRNAs with spatiotemporal expression patterns in the skin of goat during the cashmere growth cycle
Source: BMC Genomics. 2018 Jun 26;19:495. doi: 10.1186/s12864-018-4864-x (PMC6019838; doi:10.1186/s12864-018-4864-x)

Figure S1

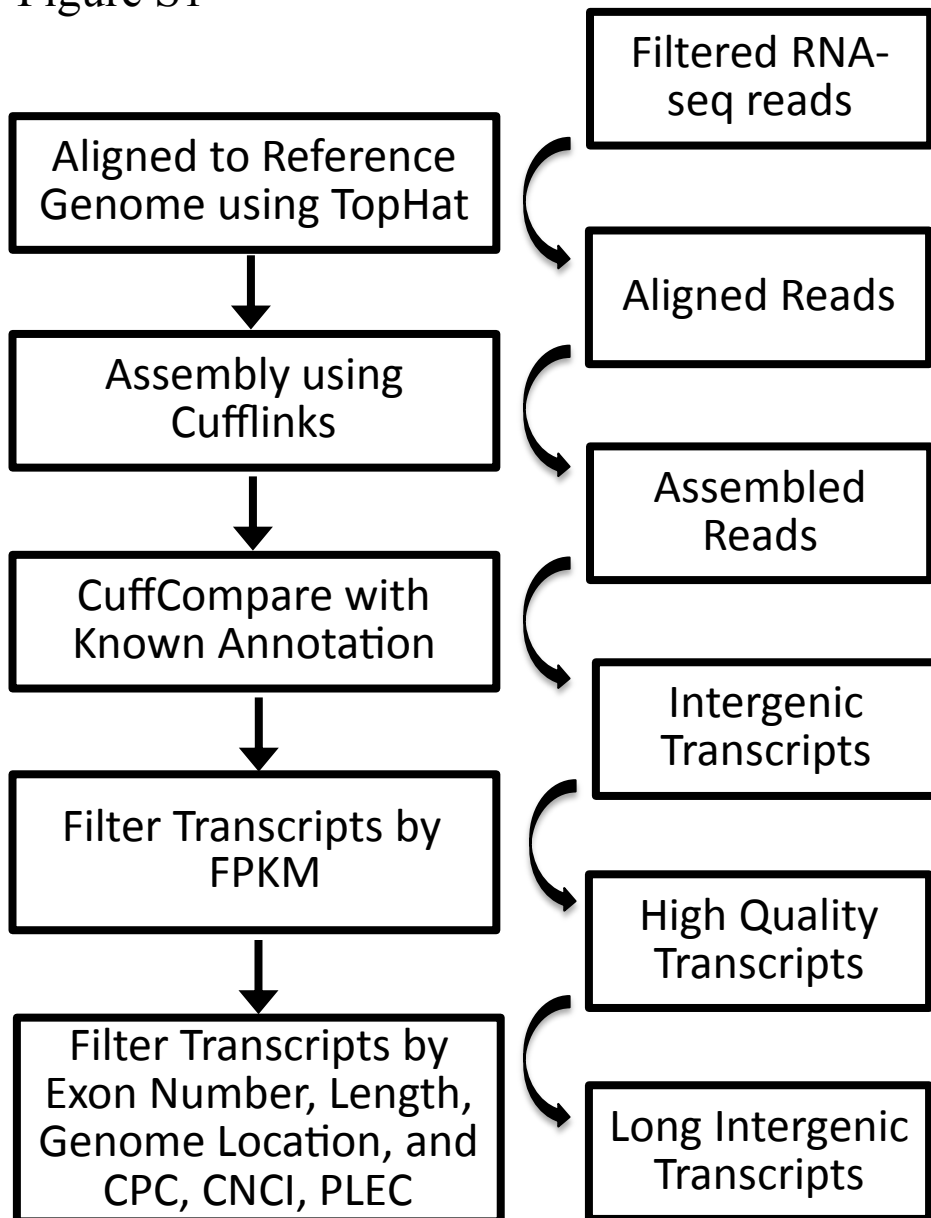

Supplement: Supplementary file 2 — Figure S1. Overview of the informatics pipeline used to the identify lincRNAs in goat. (PDF 216 kb) [file 12864_2018_4864_MOESM2_ESM.pdf]

Figure S2

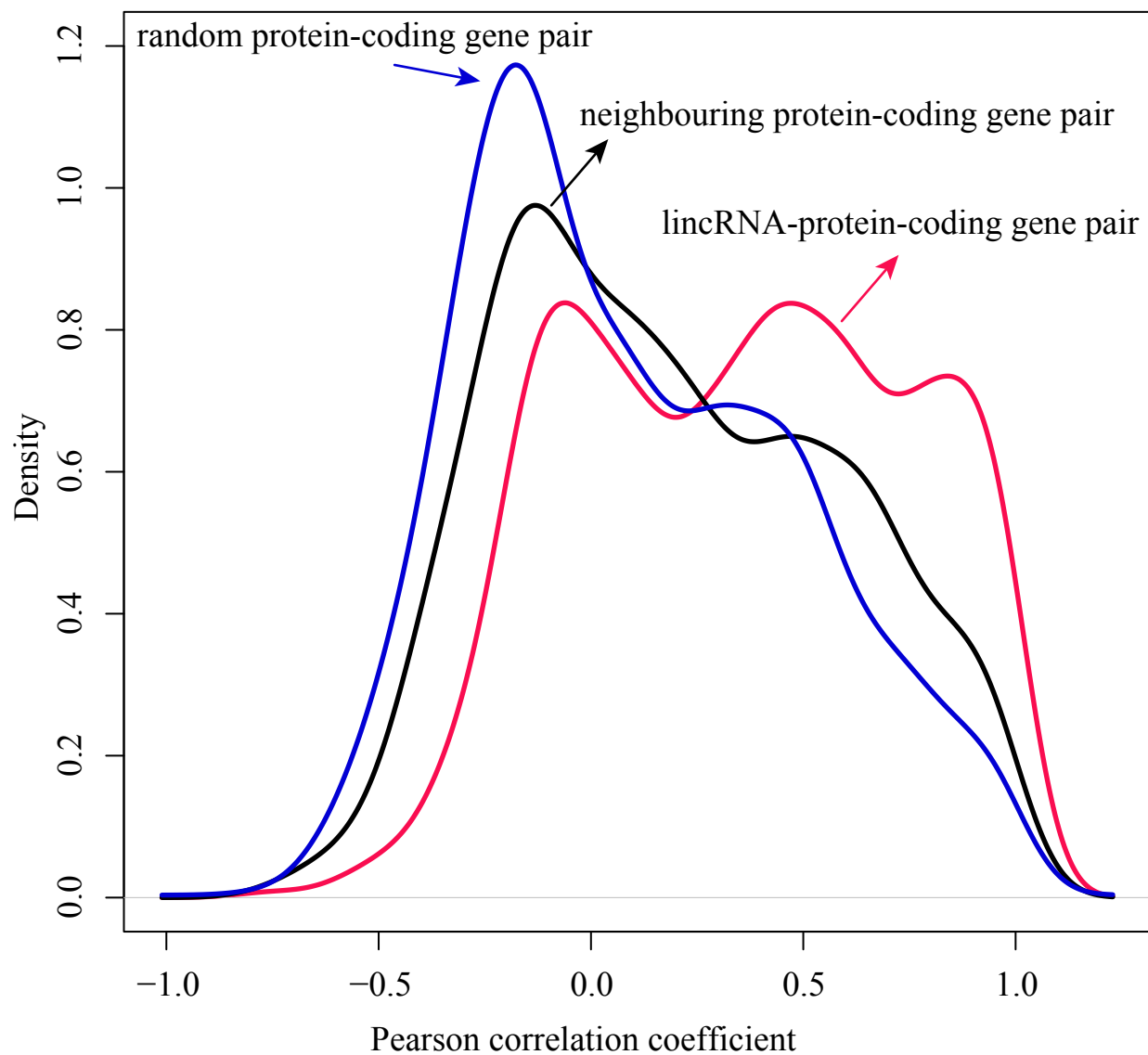

Supplement: Supplementary file 3 — Figure S2. Density plot of Pearson correlation coefficient. (PDF 151 kb) [file 12864_2018_4864_MOESM3_ESM.pdf]

Figure S3

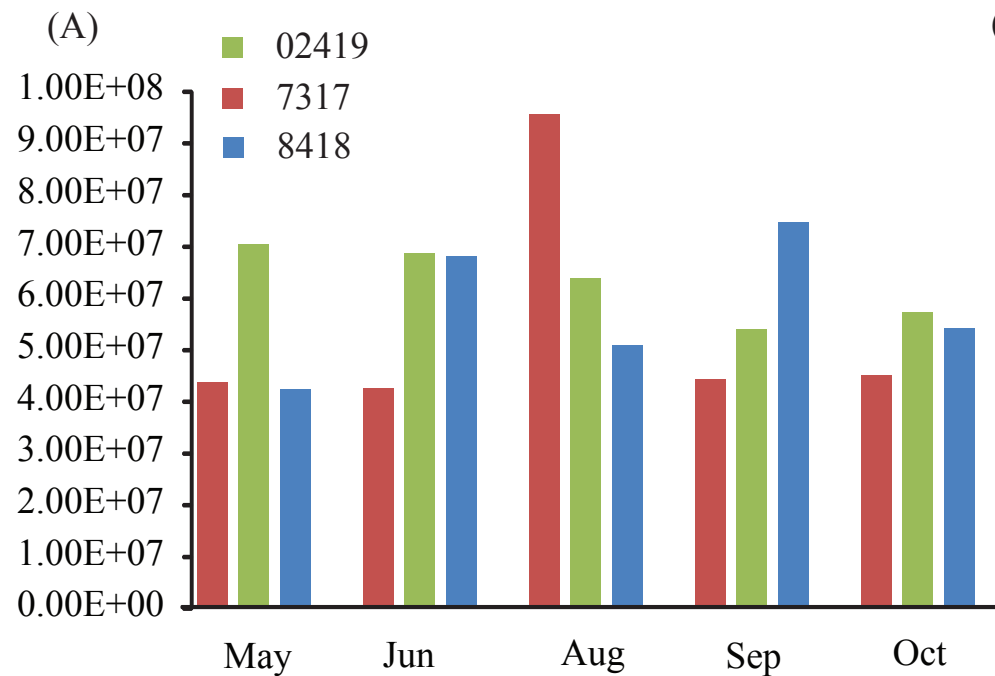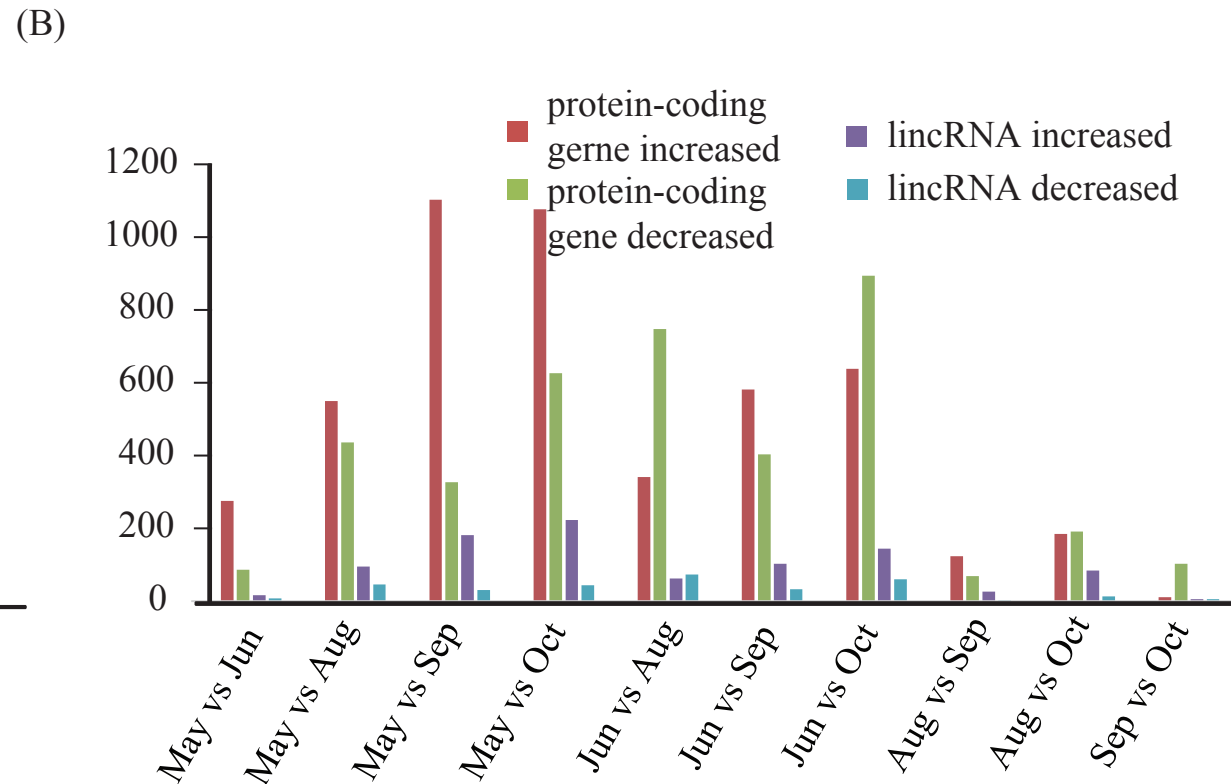

Supplement: Supplementary file 4 — Figure S3. Characterization of the HF development-related gene expression in skin. Note: A. Number of RNA-seq reads obtained from the 15 samples at five time points. B. Histogram presenting differentially expressed genes with an adjusted P value ≤0.05 for at least one of the ten comparisons (May vs. Jun, May vs. Aug, May vs. Sep, May vs. Oct, Jun vs. Aug, Jun vs. Sep, Jun vs. Oct, Aug vs. Sep, Aug vs. Oct, and Sep vs. Oct). (PDF 275 kb) [file 12864_2018_4864_MOESM4_ESM.pdf]

FigureS4

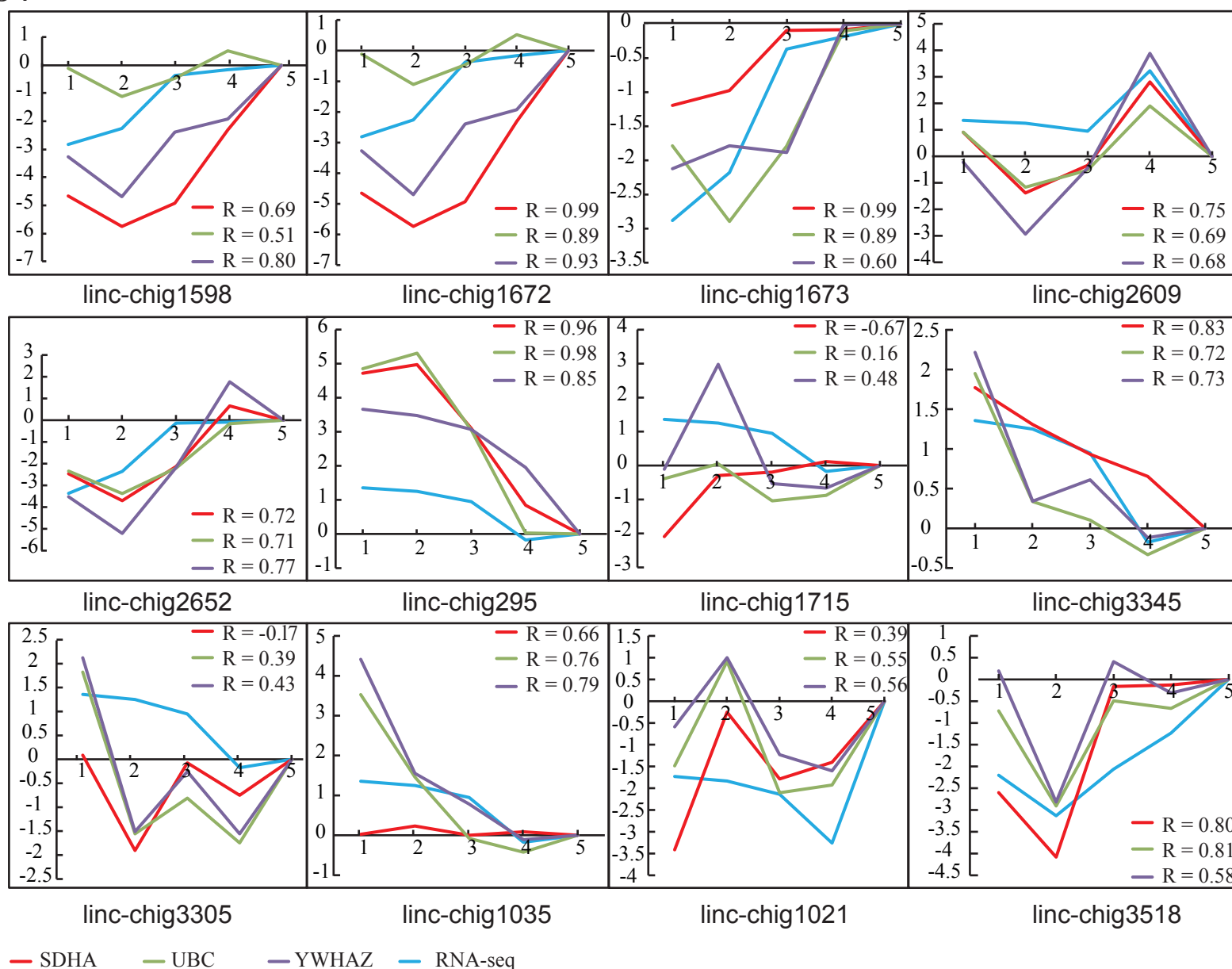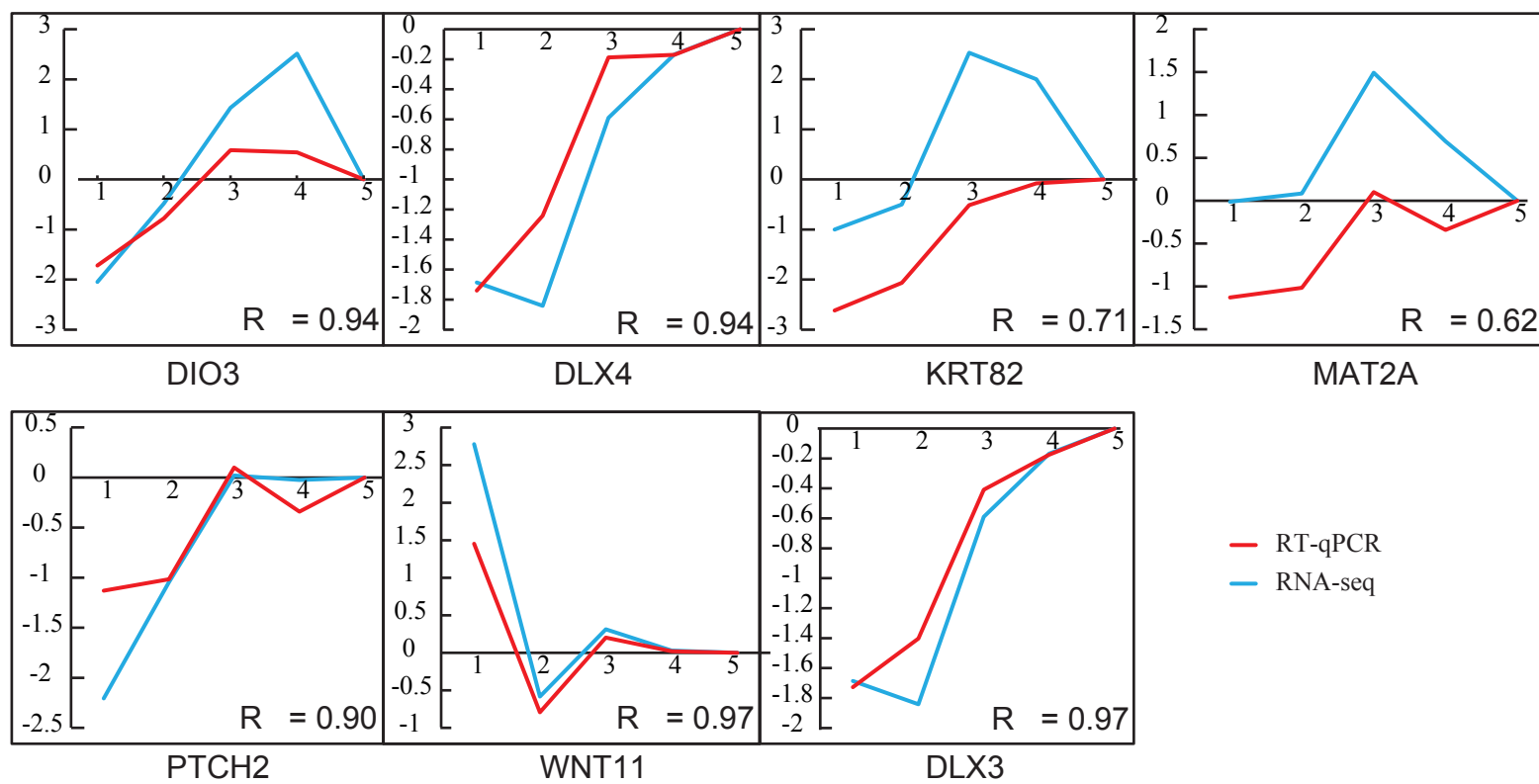

Supplement: Supplementary file 5 — Figure S4. Comparison of the expression patterns of selected lincRNAs and protein-coding genes detected in RNA-seq (red line) and RT-qPCR (blue line) assays revealing a high correlation between the two methods. Note: The log2 ratios of the expression changes during Oct relative to the other time points were calculated and plotted (the ratio was set to 0 for the normal condition). The expression levels of the selected protein-coding genes were normalized against that of β-actin. The expression levels of the selected lincRNAs were normalized against that of SDHA, UBC and YWHAZ. The R values (Pearson correlation coefficients) across the different time points are presented for each gene. (PDF 543 kb) [file 12864_2018_4864_MOESM5_ESM.pdf]
